# Lentiviral Transduction of CD34^+^ Cells Induces Genome-Wide Epigenetic Modifications

**DOI:** 10.1371/journal.pone.0048943

**Published:** 2012-11-07

**Authors:** Yoshiaki Yamagata, Véronique Parietti, Daniel Stockholm, Guillaume Corre, Catherine Poinsignon, Nizar Touleimat, Damien Delafoy, Céline Besse, Jörg Tost, Anne Galy, András Paldi

**Affiliations:** 1 Inserm, U951, Genethon, Evry, France; 2 Ecole Pratique des Hautes Etudes, UMRS_951, Genethon, Evry, France; 3 Department of Obstetrics and Gynaecology, Yamaguchi University Graduate School of Medicine, Ube, Japan; 4 Université Evry Val d’Essonne, UMRS_951, Genethon, Evry, France; 5 Centre National de Génotypage, CEA – Institut de Génomique, Evry, France; 6 Fondation Jean Dausset- CEPH, Paris, France; RWTH Aachen University Medical School, Germany

## Abstract

Epigenetic modifications may occur during *in vitro* manipulations of stem cells but these effects have remained unexplored in the context of cell and gene therapy protocols. In an experimental model of *ex vivo* gene modification for hematopoietic gene therapy, human CD34^+^ cells were cultured shortly in the presence of cytokines then with a gene transfer lentiviral vector (LV) expected to transduce cells but to have otherwise limited biological effects on the cells. At the end of the culture, the population of cells remained largely similar at the phenotypic level but some epigenetic changes were evident. Exposure of CD34^+^ cells to cytokines increased nuclear expression of epigenetic regulators SIRT1 or DNMT1 and caused genome-wide DNA methylation changes. Surprisingly, the LV caused additional and distinct effects. Large-scale genomic DNA methylation analysis showed that balanced methylation changes occurred in about 200 genes following culture of CD34^+^ cells in the presence of cytokines but 900 genes were modified following addition of the LV, predominantly increasing CpG methylation. Epigenetic effects resulting from *ex vivo* culture and from the use of LV may constitute previously unsuspected sources of biological effects in stem cells and may provide new biomarkers to rationally optimize gene and cell therapy protocols.

## Introduction

The *in vitro* manipulation of stem cells or embryos is now successfully employed in various fields of experimental biology and medicine. Yet, transgenesis and reproductive technologies have provided clear examples that *in vitro* culture conditions can cause environmentally-induced epigenetic changes in the manipulated cells, such as modifications in gene imprinting and DNA methylation [1], [2]. Recent studies suggest that such changes may have long-term effects on the resulting organisms [3], [4], [5]. The *in vitro* manipulation of somatic stem cells is also used in regenerative medicine, for instance in *ex vivo* cell and gene therapy strategies. *Ex vivo* gene-corrected autologous hematopoietic stem cells (HSC) are currently tested in early-phase trials to treat severe genetic diseases (for a review see [6]). This approach consists in stable gene modification of the patient’s autologous HSC during a short *ex vivo* culture in the presence of early-acting hematopoietic cytokines. To be successful, this process must preserve the biological function of gene-corrected HSC such as their engrafting capacity, long-term self-renewal or multi-lineage potency. In general, the prolonged culture of CD34^+^ cells leads to cell differentiation. Loss of multilineage engraftment and reduced capacity for production of naïve T cells is already evident after 4 days of culture of bone marrow CD34^+^ cells in a dog model [7]. Cell proliferation and differentiation are associated with genome-wide epigenetic remodeling. In particular, DNA methylation plays an important role in HSC self-renewal, commitment to the lymphoid or myeloid lineages and in progenitor cell aging [8]. It is therefore a basic requirement that the process of HSC gene modification during *ex vivo* culture protocols should avoid cell differentiation and minimally impact on the normal epigenetic regulation and methylation profile of the cells. At present, Moloney Leukemia Virus (MLV) and human immunodeficiency 1 (HIV-1) are the two most commonly-used retroviruses for stable gene transfer into HSC in *ex vivo* applications. In this context, HIV1-derived LV present advantages over MLV vectors because HIV does not require cell division to integrate into the nucleus and shorter transduction protocol can be used [6]. LV gene-modifed HSC have been successfully used in clinical gene therapy protocols in humans, leading to the sustained engraftment of multilineage gene-corrected blood cell [9]. Although the optimization of the HSC gene modification process is a central issue in gene therapy, the question of epigenetic consequences due to the *ex vivo* culture including the use of the vector, has never been directly explored.

While it is known that the epigenetic status of target cells has an impact on the integrated expression cassette in several ways, it is not known if gene transfer vectors can induce epigenetic changes in cells. Retroviral vector integration requires the assembly of a preintegration complex (PIC), containing the reverse transcribed proviral DNA and a combination of viral and cellular proteins capable of entering the cell nucleus and interacting with nuclear proteins to integrate the proviral genome into the chromatin. The genomic integration site selection of retroviruses is non-random and determined in part by virus-specific components of the PICs and poorly-characterized chromatin-binding proteins. In CD34^+^ cells, MLV and HIV1 vector genomic insertion site preferences correlate with the chromatin state and methylation status of the cells [9], [10], [11]. Both MLV and LV favor insertion in transcriptionally-active units and disfavor insertion near silent or heterochromatic loci marked by H3K27me3 and H3K9me3 histone modifications [12]. LV shows preferences for H3K36me3 histone modifications. Thus, it is important to determine if the transduction protocol may influence the epigenetic landscape of the target cells not only in the context of cell differentiation but because it might define vector insertions and biological activity of the integrated transgene.

It is conceivable that retroviral vectors themselves could potentially influence the status of the host cell chromatin although this has never been reported. In other systems, virus-associated DNA methylation changes occur for example following EBV infection of B cells [13]. Several viral-encoded enzymes in particular in adenoviruses, can have genome-wide gene effects independently of the integration through post-translational modifications of histones or other cellular proteins important for chromatin function [14], [15], [16]. Long range and widespread interactions between the virus and its integration machinery are also possible, but have not been investigated.

Therefore, the main objective of the present work was to search for evidence of epigenetic changes occurring during the transduction protocol as a result of cell culture and/or virus effects and which would define the properties of the cells used for HSC-based gene therapy. We followed a typical protocol used for LV transduction of CD34^+^ cells based on 24 hrs cytokine stimulation followed by two consecutive incubations with the LV during the following 24 hr period. Polybrene, a polycation, was added to the culture medium to facilitate the entry of the viral particles in the cell. Cells were analysed immediately at the end of the transduction protocol to measure changes in known epigenetic markers and in DNA methylation during the various steps of transduction. Our findings show that the process of LV transduction induced broad epigenetic changes and found effects associated to the LV that exceeded several fold the effects of cytokine or polybrene effects on the cells.

## Results

### Characterization of the Experimental Groups

**Figure 1:**
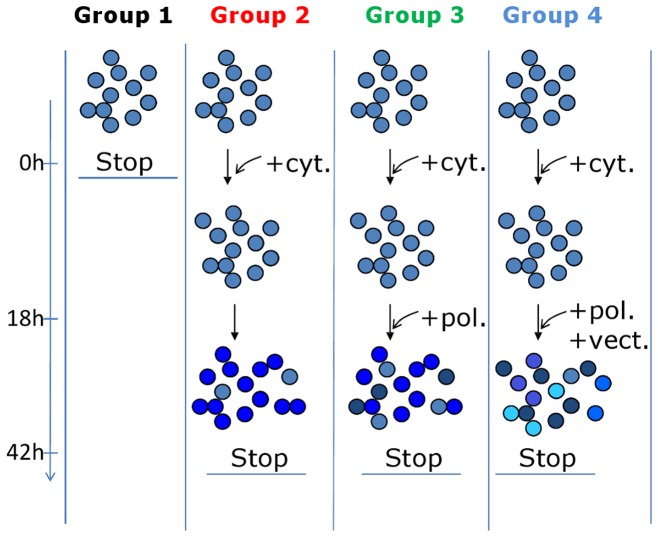
Schematic outline of the experimental strategy. Freshly isolated CD34^+^ umbilical cord blood cells were separated into four experimental groups: Group 1: non treated CD34^+^ cells analyzed immediately; Group 2: cells stimulated with cytokines only; Group 3: cells cultured with cytokines and undergoing a mock infection with polybrene only but no LV; Group 4: cells cultured with cytokines, then transduced with polybrene and LV. The cells were either fixed for immunochemical analysis or were frozen for DNA extraction and methylation analysis. In a typical experiment, cells isolated from the same cord blood were split into the different groups. For immunochemical analysis groups of cells deriving from a single cord were compared. For methylation analyses, DNA from several cord blood donors was pooled.

A model of *ex vivo* gene therapy has been developed using umbilical cord blood CD34^+^ cells transduced with a LV encoding GFP. As is typically used in clinical or experimental models, a cocktail of early-acting cytokines was added at the start of the culture to pre-activate CD34+ cells prior to transduction. Serum-free medium was used to limit influence of serum-derived factors and polybrene, a cationic polymer, was added to facilitate LV transduction of CD34+ cells [17]. Following preactivation, the LV was added twice consecutively. This protocol leads to the stable expression of GFP in 53% +/−15% (n = 37 cord blood donors) of cells in culture as measured by FACS one week following infection with the LV and continuous culture of cells with cytokines. This particular protocol maintains the multipotent differentiation capacity of transduced cells as measured *in vitro* in clonogenic assays [17] and *in vivo* by the detection of various lineages of cells following reconstitution of immunodeficient mice (not shown). To measure what changes are induced in the cells by the different steps of the transduction process itself, independently from subsequent cell expansion and differentiation, we designed the protocol shown in Figure 1.

Four conditions were compared. Group 1 consists of non treated CD34^+^ cells taken at the start of experiment to serve as reference point. Groups 2, 3 and 4 are parallel cultures of CD34^+^ cells, which compare incrementally the additive effects of cytokines, polybrene and LV on the cells. Total time of infection with the LV was 24 hours and total time of the *ex vivo* cell culture process was about 42 hours.

**Figure 2:**
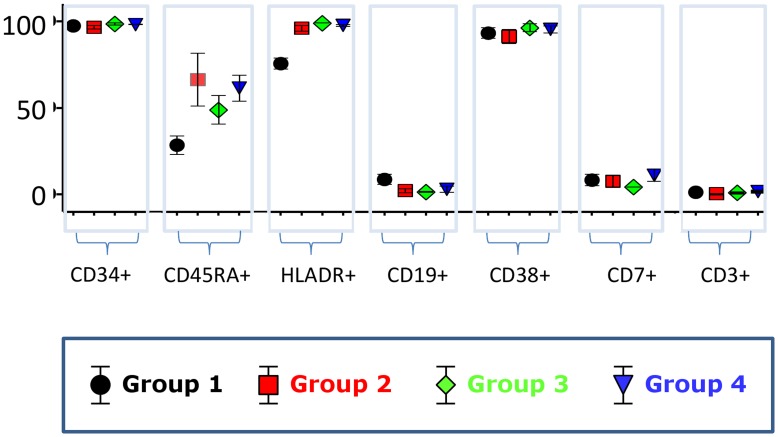
Flow cytometry analysis of the CD34^+^ cells at the end of the 42 culture period. 5 different UCB samples were analysed. Group 1 to 4 were defined as on Figure 1. The box-plots show the fraction (+/− SD) of cells positive for the analysed cell surface markers in the four groups. Note that HLA-DR and CD45RA increased in the cytokine-stimulated as compared to the unstimulated cells, but the other markers remained stable. No effect of the LV and/or polybrene treatment could be detected.

This short transduction process does not modify the proportion of CD34^+^ cells in the culture. This marker remained high in the cell population in all conditions as detected by FACS analysis at the end of the experiment (Fig. 2). No lineage-specific differentiation marker such as T or B cell markers was acquired during this short period. However, subtle changes were observed as HLA-DR and CD45RA levels increased in the cultured cells. These changes were induced by cytokines, and no additional change was observed in response to polybrene or lentiviral vector addition in the culture.

### Immunofluorescence Analysis

**Figure 3:**
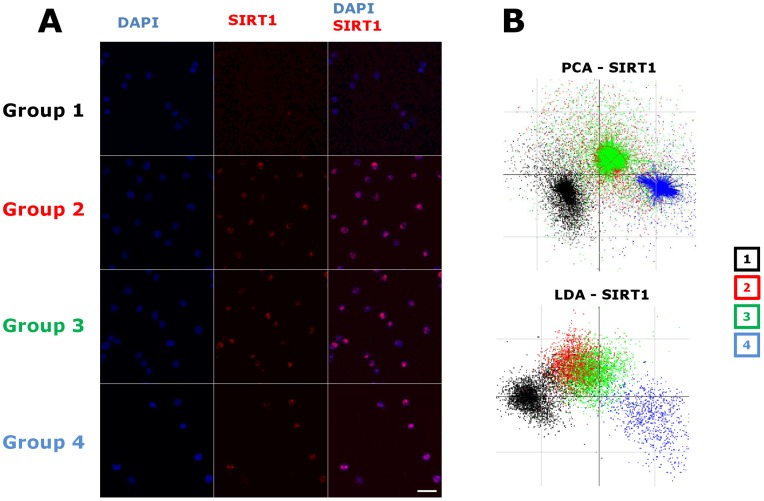
High-content image analysis of the heterogeneous population of SIRT1 immunostained cells of the four groups suggests changes in the cell nucleus. **A.** Examples of images used for the analysis of SIRT1 expression representative of 9 experiments. The groups and colour codes are the same as indicated on the Figure 1. Scale = 50 micrometers. Confocal analysis was performed on a LEICA TCS SP2 microscope (Leica Microsystems) with the Leica Confocal Software. Images were acquired with a 40X HCX PL APO Plan Fluor oil objective (NA 1.25) at room temperature. For display, the images were processed using ImageJ a median filter 3 pixels followed by an image adjustment, increasing the brightness was performed equally on all the Red images. The nuclei were stained with DAPI and the SIRT1 antibody was labelled with Alexa 594 **B.** Upper plot: A representative example of the principal component analysis of the data extracted from the SIRT1 immunostaining images. Individual cells are visualized as points on the scatter plot of the first two principal components describing the variation. They were identified by the colour code according to their group of origin only after their position on the scatter plot is calculated. Note that cells from the same experimental group tend to cluster. The groups 2 and 3 overlap. Lower plot: Representative example of the LDA analysis of the same data. This alternative multiparametric analysis takes into account the existence of four groups and helps to visualize the differences between them. Note the overall similarity of the LDA and PCA scatter plots.

**Figure 4:**
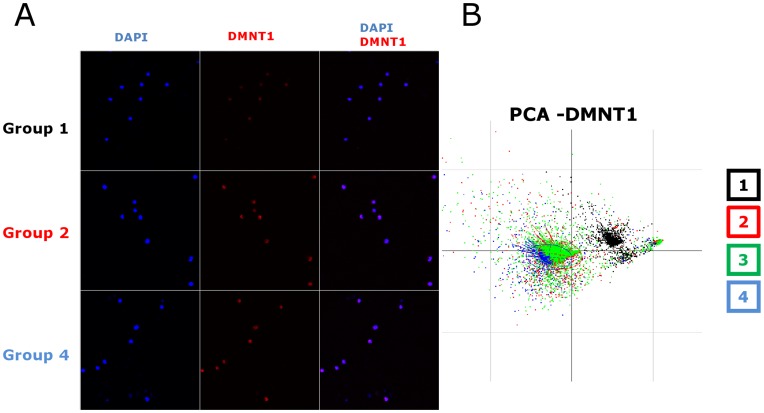
High-content image analysis of the heterogeneous population of DNMT1 immunostained cells of the four groups suggests changes in the cell nucleus. **A.** Examples of immunostaining images used for the analysis of DNMT1 expression representative of 9 experiments. The experimental and image analyse procedures were the same as on Figure 3. **B.** Representative example of the PCA analysis of the data extracted from analysis of the images of DNMT1 immunostained cells. Note that the cluster of cytokine-stimulated and virus-transduced cells overlap.

**Figure 5:**
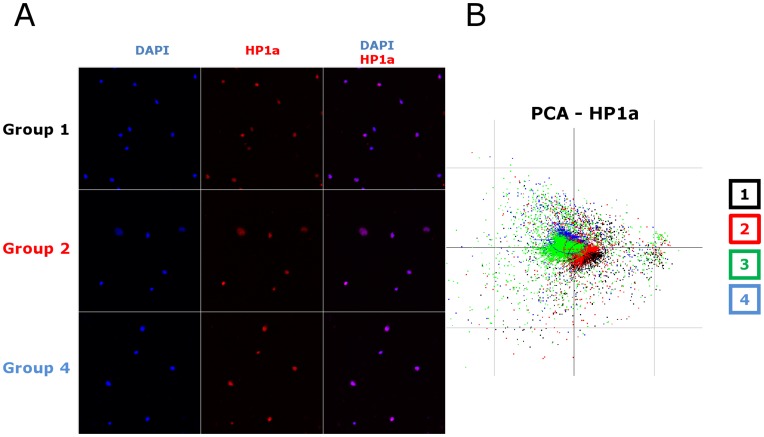
High-content image analysis of the heterogeneous population of HP1alpha immunostained cells of the four groups suggests changes in the cell nucleus. **A.** Examples of immunostaining images used for the analysis of HP1alpha expression. **B.** Representative example of the PCA analysis of the data extracted from analysis of the images of HP1alpha immunostained cells. Note that the three clusters overlap.

To measure if alterations were induced in the cell nucleus at the level of the chromatin, we analyzed nuclear patterns of certain proteins known to be involved in epigenetic processes. The method is based on the immunofluorimetric detection of the nuclear proteins chosen for their role in the establishment and maintenance of various aspects of epigenetic patterns of the genome. Class III histone deacetylase SIRT1, DNA methyltransferase 1 (DNMT1) and Heterochromatin Protein 1alpha (HP1alpha) were detected using monoclonal antibodies, followed by automatic acquisition and computer-based quantitative analysis of images of a large number of cells (2000– to 3000 cells) in each group, submitted to automatic image processing. Similar image analysis-based methods has been successfully used in various experimental studies to characterise the heterogeneity of cell populations and the effects of various external factors [18]–[23]. Results for SIRT1 are shown in Figure 3, for DNMT1 on Figure 4 and for HP1alpha on Figure 5. The DAPI staining was used for image segmentation and identification of the cell nuclei. The established nuclear mask was used for the pixel-by-pixel quantification of the nuclear protein immunostaining intensity. Nuclear shape features and features describing the texture and intensities of immunolabeling were extracted from these values for each cell in each group. For a given type of immunolabeling, the datasets obtained from the different cell groups in the same experiment were merged. None of the individual parameters alone were sufficient to differentiate the experimental groups. Thus, to determine whether potential differences exist or not between the different experimental groups, we performed principal component analysis (PCA). This mathematical procedure reduces the large number of features extracted from the fluorescence values to a few artificial variables, the principal components (PC-s), which account for most of the variability in the original data and can be visualized on a two-dimensional plot. In the case of SIRT1 immunolabeling, visual microscopic examination showed a clear induction of the protein in the nuclei of activated cells (Fig. 3A) but this approach did not distinguish an effect of polybrene or LV on the nuclear patterns of SIRT1 in the cells. In contrast, using automated image analysis the PC1/PC2 plot of the first two principal components showed three different clusters that matched with the cell populations included in the analysis (Fig. 3B): the control unstimulated cell group (Group 1) formed one cluster, the cytokine stimulated (Group2) and cytokine stimulated in the presence of polybrene cell (Group 3) clusters overlapped and the transduced cell population formed a clearly distinct cluster. Statistical analysis (MANOVA) of the original data obtained from the cell populations indicated a highly significant difference between them (p<10^−16^) suggesting that the nuclear SIRT1 pattern reflects structural differences in the nuclei between the cell populations. The PCA was completed by linear discriminant analysis (LDA) that allows the best discrimination between the groups. As shown on Figure 3B, the four cell populations appeared clearly as separate clusters.

Whereas the SIRT1 staining allowed clear distinction between the four cell populations, other markers were not as discriminatory even though they were found to be expressed in activated CD34^+^ cells. Principal component analysis of DNMT1 expression discriminated between the quiescent and cytokine-stimulated CD34^+^ cells. The DNMT1 protein was induced by cytokine stimulation, but no difference was detected that could be attributed to LV transduction upon visual inspection (Fig. 4A or using PCA or LDA (Fig. 4B). The detection of HP1alpha did not discriminate any of the populations whether activated or not, transduced or not (Fig. 5). Thus, the augmented intensity and changes in nuclear distribution of SIRT1 and DNMT1 presumably reflect the epigenetic changes ongoing upon cytokine stimulation and which is known to induce cell cycling and initial stages of cell differentiation. SIRT1 nuclear staining pattern is also modified by LV. Since SIRT1 is a major histone deacetylase, these changes are strong indications that epigenetic differences are also induced by LV beyond the effects of cytokines. Nevertheless, the technique does not provide information about the nature of these epigenetic changes.

### Study of Genomic Methylation

**Figure 6:**
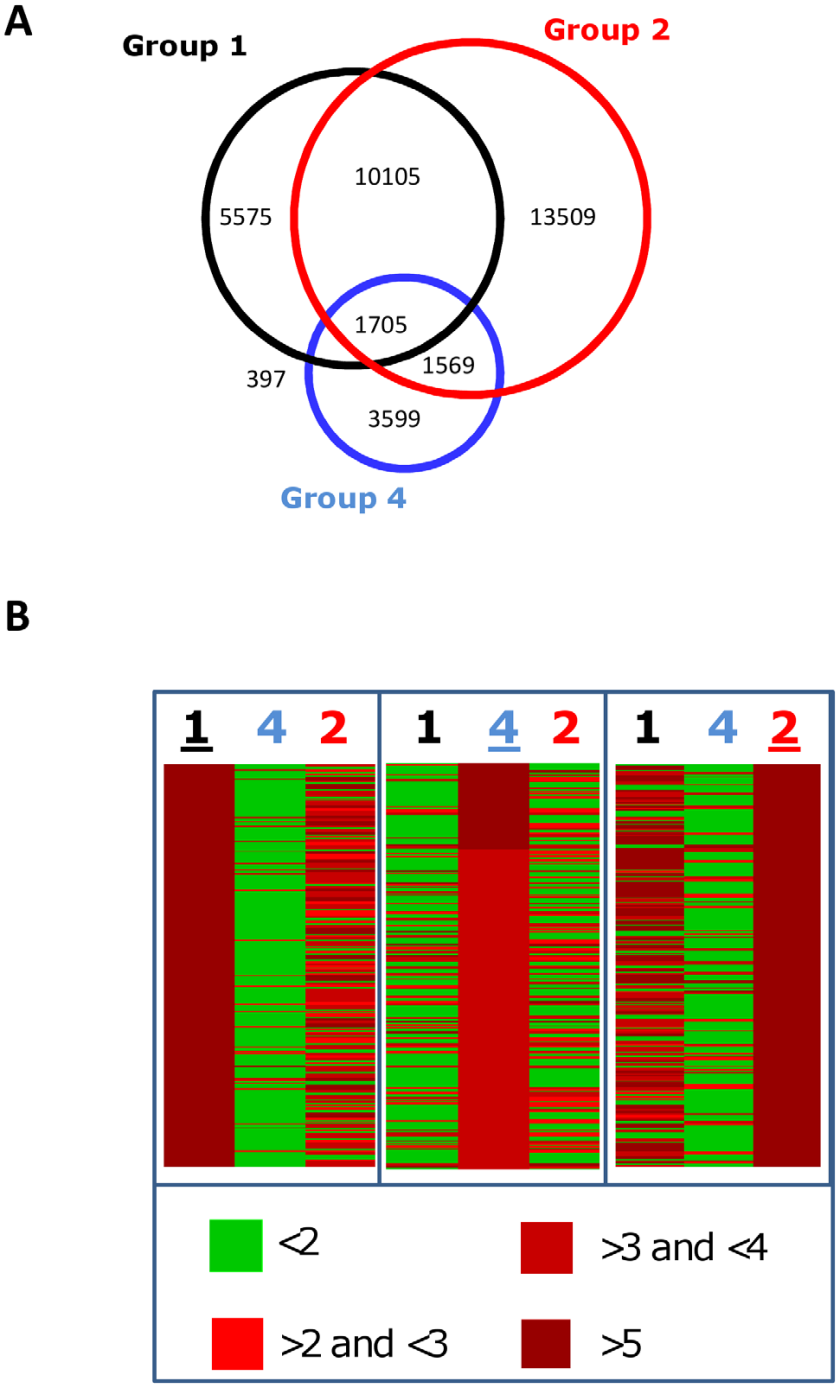
Lentiviral induced DNA methylation changes detected by MeDIP analysis. A . Venn diagram representing the sets of tags with log_2_ ratios >2. Each group is represented by a circle: non-stimulated CD34^+^ cells cells (Group 1; black circle), cytokine pre-stimulated (Group 2; red circle) and LV transduced (Group 4; bleu circle). Intersections represent the tags shared by two or three groups. A color code is used to designe the unique sections and the intersections and the number of tags in each section are indicated on the right side. As a result, the total number of tags identified in a group is equal to the summ of the four sections that fill up the circle. The little overlap between the three groups suggests that the difference in the methylation is extensive. **B**. Heat-map comparison of the tags with the highest log_2_ ratios in the different groups. Left panel shows the first 500 tags with the highest log_2_ ratios in Group 1 and the corresponding log_2_ ratio in the two other groups. The middle panel shows the 500 tags with the highest log_2_ ratios in Group 4 together with the corresponding values in the other groups. On the right panel the log_2_ ratios of the first 500 tags in the Group 2 are compared to the other groups. The color code of the log_2_ ratios is indicated under the panels. Note that most of the tags with the high log_2_ ratio in one group have a different log_2_ ratio in the two other groups. The greatest difference is observed between the Group 1 and 4.

Genomic CpG methylation is a prominent epigenetic modification that is involved in long-term regulation of gene expression. Two independent methods were used to compare the methylation profiles of CD34^+^ cells upon transduction. First, we performed immunoprecipitation of methylated DNA fragments of extracted genomic DNA (MeDIP), followed by hybridization on a genomic tiling array (3×720 K NimbleGene) covering known gene promoters and CpG islands. We analyzed DNA methylation in Groups 1, 2 and 4 that were also compared by the immunofluorescent image analysis-based approach. We considered tags with a log_2_ peak ratio of the hybridization signal for the precipitated/input DNA fraction higher than 2. Their analysis revealed substantial and widespread differences in the genomic methylation patterns of the different groups tested (Fig. 6). The highest number of tags with a log_2_ peak ratio >2 was found in cytokine-stimulated cells (26888 tags) whereas the control CD34^+^ cells displayed 17782 tags. The LV transduced cells had a distinctly different result from the other 2 groups with only 7270 tags. The total number of tags that were enriched by immunoprecipitation altogether in the 3 groups was high (36459 tags) and changes were very diverse throughout the genome since only 5% of the tags (1705 tags) were shared between the 3 conditions tested (Fig. 6A), suggesting significant differences between the methylation patterns induced by the different conditions. This is illustrated by the heat map representation of the first 500 tags with the highest log_2_ peak ratio, showing that distinct patterns were observed among each of the groups (Fig. 6B). As seen in this representation, most of the differences were observed between the LV transduced cells and the two other groups (group 4 versus 1 or 2), while there were fewer differences between the control unstimulated and cytokine-stimulated cells (group 1 versus 2).

MeDIP is based on the relative enrichment of methylated DNA fragments after immunoprecipitation. This method is not quantitative; the log_2_ peak ratios do not reflect the quantitative methylation value. Although the antibody enrichment correlates in a non-linear fashion with the methylation level, the enrichment also depends on the CpG-density and distribution within a given region [24]. Thus, the difference of the log_2_ peak ratio value of the same marker between different samples reveals changes in the methylation level and/or pattern of this marker. Therefore, the high number of makers with changing log_2_ peak ratios seen in the LV transduced cells compared to the non-transduced cells certainly reflects substantial alterations in DNA methylation between the studied populations and suggests that these epigenetic changes are widespread in the genome but provides little information at the quantitative level.

**Figure 7:**
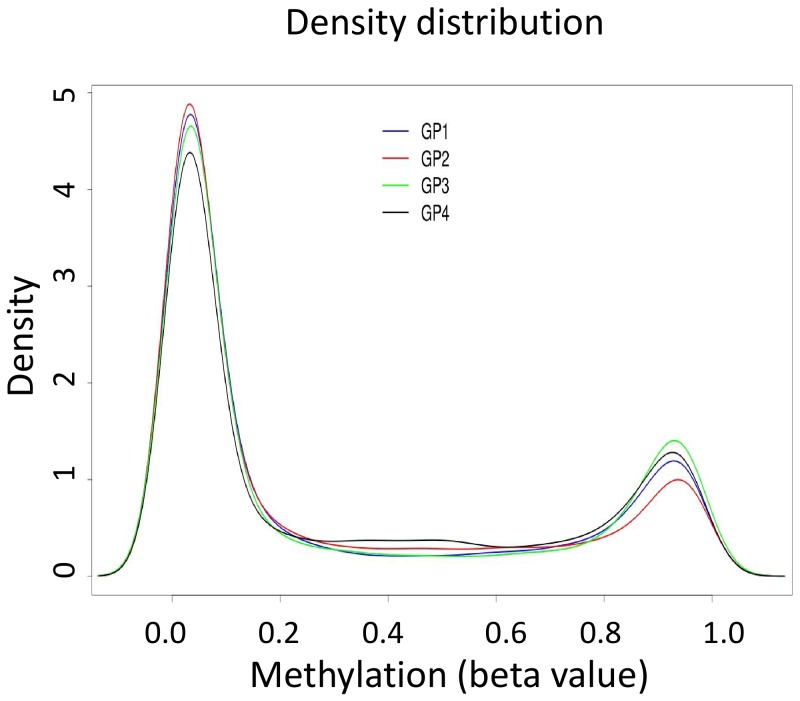
Average methylation profiles (beta values) obtained for the CpG-s analysed in each experimental condition. Each density plot represents the median of three samples.

**Figure 8:**
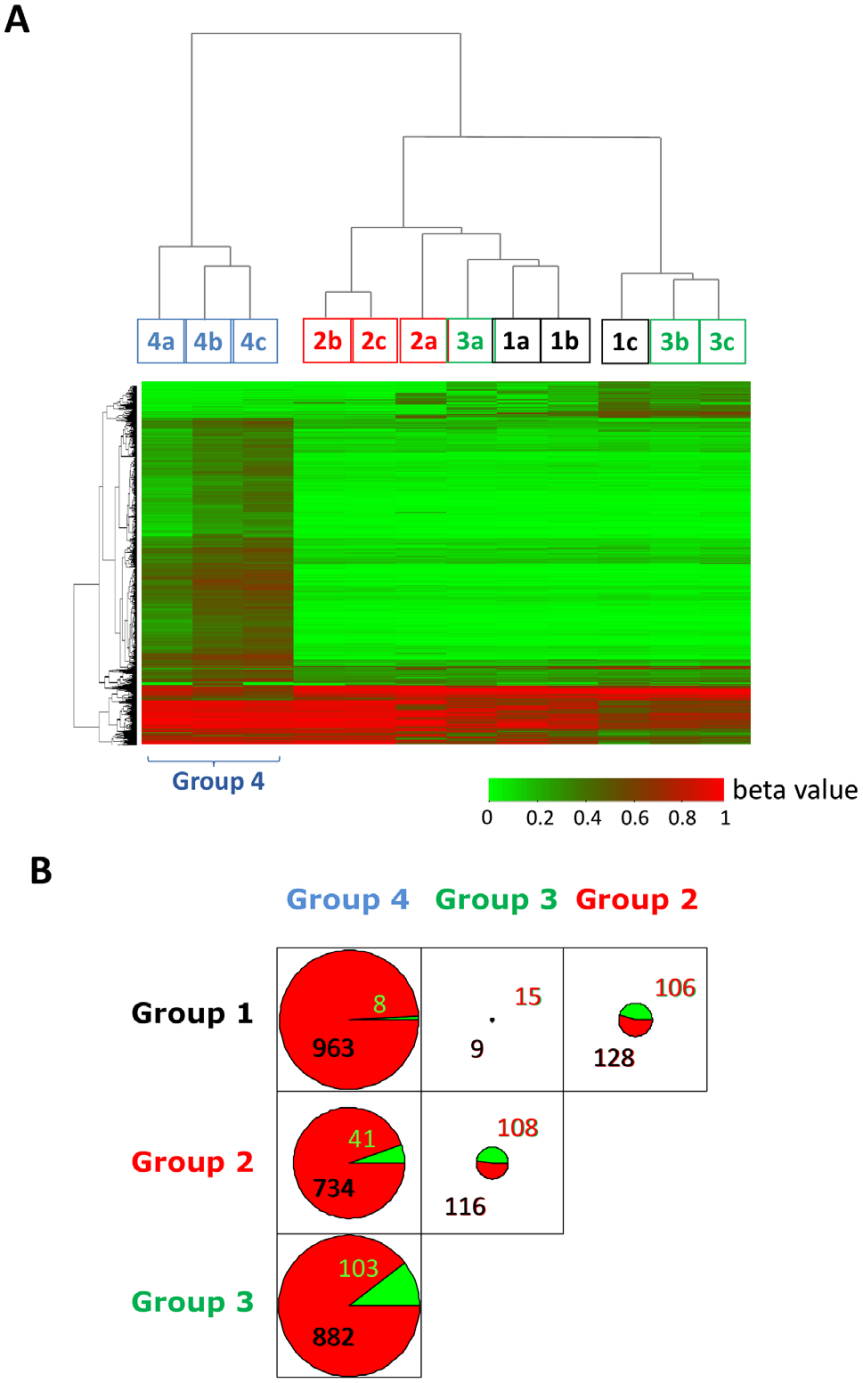
Illumina Infinium analysis also detects distinct methylation patterns in the CD34^+^ cells depending on the stimulation and/or lentiviral transduction. **A**. Dendogram obtained by unsupervised hierarchical clustering of 12 samples (triplicates for each condition) analysed. The LV transduced cells (Group 4) clusters separately of all others (upper part). The heat-map columns under the squares with the sample number represent the methylation level (beta values) of the genes that changed by at least by 20% between the experimental conditions. **B**. Comparison of the numbers of genes that increased (red) or decreased (green) methylation between the groups. The number of methylated/demethylated genes increases dramatically in LV transduced as compared to the two the non-transduced CD34^+^ cells.

Therefore, to confirm that extensive alterations of methylation are induced by LV and to quantify these changes, we used an alternative large-scale Illumina Methylation Assay which uses microarray hybridization and single-base extension on bisulfite converted genomic DNA. This method measures quantitatively the methylation level at 27,578 individual target CpG sites located in almost 14000 genes. We analysed independent triplicates of the four groups of cells described in Figure 1. Analysis of the distribution of the methylation scores (beta-values) revealed (Fig. 7) that the samples from the LV transduced cells showed moderate enrichment in intermediately methylated loci with simultaneous decrease of unmethylated and fully methylated sites. Unsupervised hierarchical clustering of the 12 methylation profiles revealed that the DNA from the LV transduced cells displays remarkable differences compared to all other samples (Fig. 8A). The heatmap representation of the genes that display substantial methylation change (at least 20% in the beta-value) shows that the LV transduced cells methylated a large number of target CpG-s relative to cytokine stimulated cells (Fig. 8A). These observations indicate a substantial change of the genomic methylation profile in LV transduced cells; hence confirm the results obtained with MeDIP.

The comparison of the number of genes that display substantial (at least 20% in the beta-value) methylation differences between the different groups is also in agreement with MeDIP results. As shown in Figure 8B, cytokine stimulation leads to a general tendency of equilibrated methylation/demethylation changes in CD34^+^ cells; the methylation of CpG targets increased in 106 and decreased in 128 genes respectively. Incubation of the cells with polybrene appeared to slow down the process of epigenetic remodelling induced by cytokines. Groups 1 and 3 were very comparable since only 24 genes were found to differ by 20% of methylation level when polybrene was added in addition to cytokines and compared to the unstimulated control group. This is in line with the recent observation showing that polybrene used during lentiviral transduction inhibits the cell cycle [25]. By contrast, LV transduction significantly stimulated methylation changes in comparison to any other group of cells, with a marked tendency for augmenting CpG methylation (Fig. 8B). About 900 genes were found to undergo methylation changes as a consequence of LV transduction in comparison to other conditions. Adding the LV induced about 4 times more changes than cytokine activation. The overwhelming majority of genes gained methylation following LV addition (Fig. 8B). Altogether, the Illumina BeadChip methylation analysis showed that the LV infection increased the tendency to methylate DNA at a large number of CpG-s. Newertheless, it is difficult to deduce a general tendency for methylation change in the genome, because the Illumina 27 K array represents only a minute fraction of the CpG-s in the genome and both the timing array used in MeDip and the Illumina array are strongly biased for gene promoters and CpG islands.

**Figure 9:**
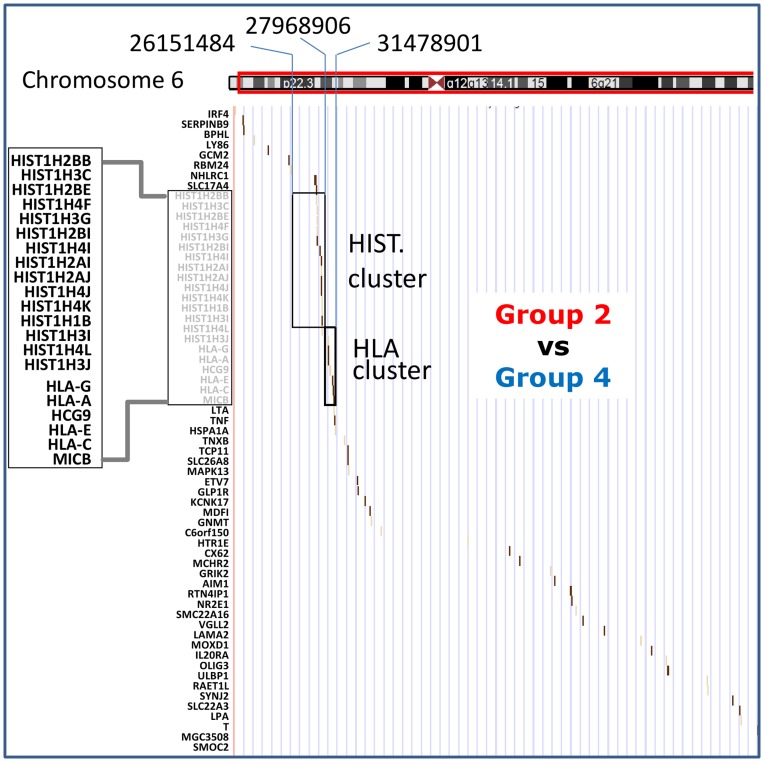
The region of chromosome 6p bearing genes methylated differentially between Group 2versus Group 4 cells. Chr 6 is schematically represented on the top. Target genes with more than 20% gain of methylation are indicated by a small vertical bar as a function of their chromosomal location and their methylation: the darker is the bar higher is the gain of methylation. Note the accumulation of the targets genes in the cluster of histone- and HLA-coding genes.

The biological pathways analysis performed with Ingenuity Pathway Analysis showed that most of the genes that significantly changed methylation (at least 20% differences in the beta-value) in response to cytokine stimulation, were linked to a functional network related to hematopoietic system function and development (Associated network functions: 1. Inflammatory response, Haematological system development, cell signalling and cell differentiation; network score = 108; 2. Cell Signalling, Molecular Transport, Vitamin and Mineral Metabolism; score = 18). This is consistent with the fact that cells were activated and stimulated to enter the differentiation pathway. Then we analysed the functional categories involved in the list of genes that changed methylation in response to the vector (group 2 versus group 4) (using DAVID tool). This analysis showed that most of the genes that underwent changes fit in the same functional categories or those related to normal cellular functions (1: Cell cycle, cellular function and maintenance, gene expression; score = 91; 2: Cellular movement, Haematological system development and function, haematopoiesis; score = 81; 3: Cell signalling, Molecular transport, Cell morphology; score = 75). This observation suggests that the methylation changes induced by the LV are not targeted to specific gene categories. Presumably, the methylation changes induced by LV treatment occur in an opportunistic way in regions with accessible chromatin structure as a result of ongoing gene regulation in the cells.Chromosomal distribution of the target genes with more than 20% differences in the beta-value in cytokine stimulated cells (Group 2) versus unstimulated (Group 1) and LV transduced (Group 4) versus unstimulated CD34 cell controls indicated that most of the changes occurred randomly with respect to their chromosomal position. However, certain chromosomal regions displayed significantly higher than random number of methylation changes (Table 1). Importantly, the same regions that beared non-random methylation changes in cytokine stimulated cells (Group 2) carried even higher number of changes in LV infected cells (Group 4). This was particularly true for the Chr 6p22.3 region hosting a large number of histone-coding genes (Table 1 and Fig. 9).

**Table 1:** Chromosomal clusters.

Group 1 *versus* Groupe 2					Group 1 *versus* Groupe 4			
chr	start	end	Nb ofGenes	pval_adj	start	end	Nb ofGenes	pval_adj
**1**	201862312	210344971	4/85	0,0497	201862312	208022283	9/68	0,0376
**2**	43717916	47467661	3/23	0,0351	42847736	45025945	5/13	0,0166
**3**	44941663	46283103	2/15	0,0478	26151484	46481657	7/28	0,0175
**6**	**17389792**	**27968906**	**7/106**	**0,0288**	**26151484**	**27968906**	**19/61**	**7.68E-7**
**7**	6696620	98901744	10/356	0,0425	86970884	89632076	4/8	0,0186
**8**	22280737	23486008	2/18	0,0497	22280737	24440371	6/24	0,0276
**11**	102146455	111261774	3/44	0,0478	110843465	111261774	2/6	0,0465
**14**	22664346	23879036	4/46	0,0351	22656387	22904682	4/8	0,0186
**19**	56940237	57021254	2/3	0,0288	56940237	57021254	2/3	0,0408

**Legend:** Chromosomal regions bearing non-random methylation changes in the genome of Group 2 (cytokine stimulated) cells (left panel) and in Group 4 (LV infected) cells (right panel) compared to the genome of control Group 1 (unstimulated) cells are indicated. Chromosomal number, the nucleotide position of the start and the end of the region of interest, the number of changes/number of analysed genes in the region and the adjusted p-value are indicated. The non-random accumulation of the changes on the chromosome 6 is highly significant. Although in the other chromosomal regions the deviation from the random pattern is at the limit of statistical significance, it is important to note that in LV infected cells the changes occur in the same regions at always higher proportion.

## Discussion

Our data provide the first evidence that a process of *in vitro* gene transfer into somatic stem and progenitor cells may influence the epigenetic landscape of the cultured cell population. In an experimental model, we describe that changes in DNA methylation occur during a relatively short *ex vivo* transduction process consisting of culturing CD34^+^ cells in medium with additives and rHIV-derived LV. Changes occurred after less than 2 days in culture and within 24 h after the first contact between the LV and the cells. The rapidity of this change may be surprising but is consistent with the dynamic nature of epigenetic changes in general and the rapid turn-over of DNA methylation in particular [26], [27]. Since the correlation between DNA methylation and the other chromatin modifications is well established [28], [29], [30], we hypothesise that the epigenetic changes occuring in response to LV are not limited to CpG methylation.

The process of *in vitro* transduction potentially involves complex effects on the cells. The experimental study design was able to distinguish discrete, nevertheless linked, effects of cell culture medium and of LV on the CD34^+^ cell population. Culturing cells with cytokines only, induced subtle changes in HLA-DR and CD45RA cell surface markers and up-regulated the nuclear SIRT1 and DNMT1 levels in the nuclei of some cells. The cytokines also induced measurable changes in genomic methylation with a balanced net effect on gene methylation levels. These observations constitute to our knowledge the first characterization of rapid epigenetic changes induced by culturing CD34^+^ cells with cytokine medium. This practice is used not only for gene transduction protocols, but also in various protocols in cell therapy based on the amplification of HSC grafts which could be concerned by these results. It is not entirely surprising that cytokine-cultured HSC undergo epigenetic changes since these conditions lead over time to the loss of HSC content as CD34^+^ cells gradually differentiate in prolonged culture. All differentiation processes are accompanied by epigenetic changes and in particular during hematopoietic differentiation as shown recently by a description of genome-wide promoter methylation during this process [8]. However since the use of cytokines and media in transduction protocols remain somewhat empirical, the data that we provide here could serve as a molecular surrogate baseline to better characterize and to minimize the effects of media and cytokines during *ex vivo* cell manipulation in various cell and gene therapy applications.

The most unexpected finding is that significantly more extensive DNA methylation changes occurred in cells that were transduced by LV than with cytokines; 775 genes were altered versus 234 found in preactivated cells. The effects of the transduction additive polybrene can be ruled out as 985 genes are altered by the addition of LV over cytokines and polybrene. Polybrene seemed to modulate the effects of cytokines, which may be linked with the recent observation that polybrene can reduce the proliferation of mesenchymal stem cells during lentiviral transduction [25]. In our system, predominantly *de-novo* methylation was observed in response of CD34^+^ cells to the LV.

Since the Illumina Methylation Assay provides quantitative measures of the methylation level at single CpG-s we can make quantitative estimations of genomic changes in the cellular population. To be stringent, we only considered genes with at least 20% change of the beta-value. This means that a change from unmethylated to methylated or *vice versa* of that particular locus is found in at least 20% of all the cells. We found around 750 genes with >20% change of the beta-value in the DNA of cells after LV transduction. We calculated that the average number of methylation changes would be around 320 per haploid genome and the minimum estimation would be 150. Under the experimental conditions used in this study the number of integrated viral genomes per cell is usually 1 to 2 copies [17]. Since the methylation changes are detectable as early as 24 hours after the first contact between the cells and the LV, and because their number exceeds largely the expected number of genomic integrations, one can deduce that many more epigenetic alterations occur in cells than those that could be directly linked to the location of the few integrated proviruses. It is also possible that epigenetic changes also occur in cells that do not become productively transduced. Further experiments are needed to address this question in greater detail.

The broad epigenetic modifications resulting from the *ex vivo* transduction of the CD34^+^ cell population may modify the cells chromatin landscape that determine the LV insertion site. Both, the gene categories and the chromosomal regions that were modified by the LV coincide with those modified by cytokines. This means that increased CpG methylation induced by the LV preferentially occurred in regions that normally undergo epigenetic remodelling as a consequence of cytokine stimulation, and suggest an opportunistic effect of the LV. If this is correct, it would be predicted that LV-induced DNA methylation changes would be different in various types of cells or following various stimuli. One stricking observation is that the frequency of methylation changes induced by LV was highly significant in the region on chromosome 6p22.2–22.1 containing histone genes (Table 1 and Fig. 9). We note that the adjacent chromosomal region containing the HLA gene cluster is also highly targeted, although the effect is at the limit of statistical significance presumably because of the low number of markers used (Fig. 9). A recent study in CD34^+^ cells reports that LV genome integration occurs at very high frequency on chromosome 6p22 in the HLA gene region [10]. More detailed mapping is needed to determine if this is a simple coincidence or a true correlation between epigenetic changes and LV integration pattern.

Our finding strongly suggests that broad epigenetic changes induced by *ex vivo* treatment of the cells by the medium with cytokines and by effects of the vector, may contribute to define the vector genomic insertion site profile. This may have consequences on the safety of gene transfer in clinical applications. Indeed, several cases of insertional genotoxicity have been reported with vector-induced clonal dominance, leukemic transformation, or dysregulation by post-transcriptional mechanisms such as alternative splicing have already been observed in hematopoietic gene therapy trials for primary immune deficiencies and beta-thalassemia [31], [32], [33], [34]. Our results suggest that conditions for the *ex vivo* gene-modification of the cells should be taken into consideration and examined for potential effects on the insertional landscapes through epigenetic effects.

At this stage it is difficult to determine the exact molecular mechanisms of the vector-induced epimutagenic process and the relative contribution of various vector components, such as the transfer plasmid, to the observed effect. Interactions between the viral proteins and chromatin-binding proteins are necessary to mediate the viral genome integration. These interactions, in turn, may interfere with epigenetic processes underlying cell activation or lineage-specific differentiation of the cells. Differentiation mechanisms are known to modify the functional state of the chromatin and ensure its transmission through cell division. Genome-wide rapid DNA methylation/demethylation reactions are part of the network of epigenetic mechanisms at work during cell differentiation, including in haematopoietic cells [8], [35]. Whether or not the LV-induced epimutagenic processes are stably transmitted or not remains to be tested in future experiments. Yet, we can speculate that several important mechanisms could be potentially involved in the direct interaction between lentiviral components and epigenetic remodeling processes. The target site preference of LV for integration has been shown to depend in part on the capacity of the viral integrase to bind a host chromatin binding protein, Ledgf/p75 (product of the PSIP1 gene) [36], [37]. The base excision repair pathway (BER) represents another potentially interesting candidate mechanism. The BER pathway is involved in the DNA methylation/demethylation cycle [38]. A recent study shows that BER pathway proteins are essential to the genomic integration of lentiviruses [39] suggesting direct interactions between the viral pre-integration complex and BER proteins. Last but not least, although the SIRT1 protein was used here only as a simple biomarker, it may be directly involved in the observed phenomenon. Indeed, SIRT1 is a histone deacetylase known to interact with a large number of nuclear proteins and mediate cellular responses to stress, including genotoxic stress – known for the activation of BER. These observations provide a coherent background for further study of the mechanisms involved in genome-wide methylation changes induced by LV transduction.

The consequences of the widespread epigenetic changes induced by LV are currently not known. Since transduced HSC retain the capacity to differentiate into multiple lineages of cells as shown *in vitro* and *in vivo* in various models, most of these alterations are likely to have no effect or disappear due to the reversible nature of the epigenetic modifications. Alternatively, the cells strongly affected by the induced epimutations may be rapidely selected out at the initial stages of differentiation. Nevertheless, some epimutations could induce subtle or qualitative or quantitative alterations in the functional properties of the cells in some cases and induce adverse effects at a later stage. Indeed, we observed that the engraftment of LV-transduced CD34^+^ cells in a humanized mouse model was sometimes lower than that of non-treated cells (VP and AG, data not shown). In a manner that may not be immediately visible, epimutations could have an impact, positive or negative, on the biological properties of the cells and of their progeny. Increased epigenetic variation is now acknowledged to contribute to various diseases such as malignant transformation [40], [41]. The issue of inappropriate epigenetic remodeling has also been recognized as a serious concern for the potential therapeutic use of induced pluripotential stem cells [42]. The emerging picture is that many long acting and mitotically stable effects induced by various physical, chemical or biological stresses are mediated by the epimutations they induce [4], [43].

Further evaluation of epigenetic changes induced by *ex vivo* manipulation of cells seems to be warranted from our results. In a wider methodological context, the identification of reliable biomarkers will be important to be able to rationally evaluate the impact of culture components on the cells. The image analysis approach used here may represent an efficient strategy as a primary screen for any epimutagenic effect in cultured cells. If the multiparametric analysis of the images of epigenetically relevant markers suggests a difference between the analyzed cell populations, more comprehensive molecular analysis can be performed to characterize them in detail.

## Materials and Methods

### Human Umbilical Cord Blood and CD34^+^ Cells Purification

Human umbilical cord blood samples were obtained according to French national law (Bioethics Law 2004/800) and under declaration N° DC-2008–776 to the French Government (Ministère de la Recherche et Enseignement Supérieur) to prepare the primary human CD34+ cells used for the study. The approval of an IRB was not needed for this study. These cord blood samples were obtained graciously and anonymously from the Louise Michel Hospital (CHSF, Courcouronnes, France) after uncomplicated births, for the purpose of biomedical research and with assent of the mother following information. From these cord blood samples, CD34+ cells were obtained following Ficoll gradient centrifugation and microbead-conjugated magnetic cell sorting according to the manufacturer’s recommendations (QBEND/10 from Miltenyi Biotec, Bergish-Gladbach, Germany). The purity of CD34^+^ cells was greater than 95% in all experiments.

### Generation and Titration of Lentiviruses

VSV-G-pseudotyped LVs were produced by transient cotransfection of 293T cells as described [17]. The transfer plasmid encodes the eGFP under control of the human PGK promoter and also contains an irrelevant control shRNA expressed from the H1 promoter [44]. The culture medium was collected over 48h, concentrated by ultracentrifugation, aliquoted and stored at -80°C until used. Vector titers (in Infectious Genome (IG)/ml were determined on HCT116 cells by qPCR as described [17].

### Transduction of Human Hematopoietic Stem Cells

Prior to transduction, CD34^+^ cells were preactivated for about 18 hours by culture in X-VIVO 20 medium (BioWhittaker Lonza, Verviers, Belgium) supplemented with penicillin/streptomycin (Gibco), 2 mM glutamax (Gibco) and cytokines (Flt-3 ligand 50 ng/ml, stem cell factor 25 ng/ml, thrombopoietin 25 ng/ml and interleukin-3 10 ng/ml) (Peprotech, Rocky Hill, NJ) at the concentration of 5×10^5^ cells per ml. Preactivated cells were then transduced by adding 5×10^7^ IG/ml of LV and polybrene (4 µg/ml, Sigma-Aldrich, St Louis, MO). Infection was repeated after 6 h and pursued overnight. The following morning, cells were washed and used for genomic DNA extraction, microscopy analysis or phenotype characterization by flow cytometry. A sample was retained and further cultured for 1 week to determined transduction efficiency based upon GFP expression.

### Phenotype Analysis by Flow Cytometry

Phycoerythrin (PE)-labeled anti-CD7 (clone M-T701) was purchased from BD Biosciences (San Diego, CA). PE-labeled anti-CD3 (clone BW 264/56), anti-CD45RA-PE (clone T6D11) and anti-CD19-allophycocyanin (APC) (clone LT19) were purchased from Miltenyi. PE-labeled anti-HLA-DR (clone Tü36) was purchased from Invitrogen and phycoerythrin Cyanin 7 (PC7)-labeled anti-CD34 (clone 581) from Beckman Coulter. Cells suspensions were FcRII-blocked (γ-globulins from human blood, SIGMA) before staining with fluorescent antibodies. Cells were acquired and analyzed with LSRII cytometer using DIVA research software (BD Biosciences). Live cells were distinguished from dead cells with LIVE/DEAD Fixable Dead Cell Stain Kits (Invitrogen).

### Immunochemical Detection and Image Acquisition

Nuclear proteins were detected by immunostaining. Briefly, 50000 cells were fixed for 5 minutes on a polylysine-coated slide using 2% paraformaldehyde solution in PBS at room temperature, washed twice in PBS and stored at 4°C in PBS. For immunostaining, the cells were permeabilized for 5 minutes at 4°C with 0.5% TritonX in PBS, washed 3 times at RT and incubated with the primary antibody for 45 minutes. The secondary antibody was applied after 3 washes in PBS for 45 minutes, and then the slides were mounted with Fluoromont/DAPI. Antibodies: rabbit anti-*SIRT1* was from Abcam (ab32441), rabbit anti-*DNMT1* from Abcam (ab19905), mouse anti-*HP1alpha* was purchased from Upstate (05–689) and the secondary antibodies were Alexa Fluor594 labeled anti-rabbit IgG (A11012) and anti-mouse IgG Alexa Fluor 488 (A11017) from Invitrogen-Molecular Probes.

Confocal analysis was performed on a LEICA TCS SP2 microscope (Leica Microsystems). Images were acquired with a 40X Plan Fluor oil objective (NA 1.3) using the 405 nm laser for the DAPI staining and the 594 nm laser for Alexa-594 nm staining. For each field, the same parameters of gain and laser power were used and two 8 bits images containing a few hundred of cells were acquired. A minimum of 3000 cells were analyzed for each studied group.

### Image Analysis

The images were processed with the free and open source image analysis software CellProfiler (http://www.cellprofiler.org). A pipeline of methods was applied to all the pair of images (available upon request). Cells were segmented using the Otsu adaptative thresholding method for the blue channel (DAPI). The identification of the primary objects was then performed automatically with a filter on the size of the objects. Intensity information and was collected from the Red channel. A total of 25 parameters of the segmented cell were calculated: 11 parameters concerning the shape (area, compactness, eccentricity, Euler number, Shape extent, form factor, major axis length minor axis length orientation, perimeter and solidity) and 14 parameters concerning the intensity (lower quartile, upper quartile, mass displacement and median of the intensity of the all segmentation and integrated, mean, median, standard deviation, minimum, maximum for both the all segmentation and the edge of the segmentation). These data were then exported into a text table with the number of line corresponding to the number of cells.

These data were then imported in R software (http://www.r-project.org/) and Principle Component Analysis (PCA) using the package Ade4 (http://pbil.univ-lyon1.fr/ade4/) as well as Linear Discriminant Analysis (LDA) were performed. Statistical analysis of obtained proportions was done using the Multiple Analysis of Variance (Manova) function in R.

### DNA Methylation Analysis (MeDIP)

Genomic DNA was prepared from Group 1, 2 and 4 cells using Nucleospin TissuXS kit (Nacherey Nagel). Two micrograms of DNA was sent to the service provider company Imagenes (www.imagenes-bio.de). Quality control, fractionation by sonication and immunoprecipitation of the methylated DNA fraction using an anti-methylcytosine antibody followed by array analysis on 3×720 K NimbleGene arrays in duplicates and data treatement was performed according to the standard protocols used by the company.

### DNA Methylation Analysis (Illumina 27 k Array)

Methylation analysis using bisulfite conversion and array analysis was performed on DNA prepared from Group 1–4 cells. One microgram of DNA was bisulphite treated using the EpiTect 96 Bisulfite Kit (Qiagen GmbH, Germany). 500 ng of bisulphite treated DNA was analyzed using the Infinium Human Methylation 27 K beadchips (Illumina Inc, CA, USA) that simultaneously interrogates 27,578 CpG loci, covering more than 14,000 genes. Typically 2 CpG sites were analyzed per gene where one CpG site is situated in the promoter region and one CpG site is in the 1^st^ exon. The samples were processed according to the manufacturer’s protocol at the genotyping facility of the Centre National de Genotypage (Evry, France). Methylation scores for each CpG site are called as ‘Beta’ values (using BeadStudio software from Illumina), that range from 0 (unmethylated, *U*) to 1 (fully methylated, *M*) on a continuous scale, and are calculated from the intensity of the *M* and *U* alleles as the ratio of fluorescent signals b = *Max*(*M*,0)/(*Max(M,*0*)+Max(U,*0+100). Bead studio software was used for the initial processing of the methylation data. Probes for which a score was missing for any array were discarded.

### Array Based DNA Methylation Profiling and Data Analysis

Raw data were normalized according to a three step process: colour bias correction, background level correction and quantile normalization. Only beta values associated to significant p-values were selected for comparing locus methylation status between the four datasets. Data from allosomal loci were excluded from further analysis to eliminate the influence of sex-specific methylation differences. Individual loci were scored as differentially methylated if the difference between median beta values was greater than or equal to 20% of methylation.

Enriched biological pathways and functional categories of differentially methylated genes were identified by Ingenuity Pathway Analysis (www.ingenuity.com) and confirmed with the online DAVID 2.0 Functional Annotation Tool (http://david.abcc.ncifcrf.gov/).

### Chromosome Enrichment Analysis

We performed a positional gene enrichment analysis using the method described by [45] for the identification of chromosomal regions that are significantly enriched in differentially methylated genes. The proportion of a chromosome in a given gene group was compared to its proportion in the total gene population represented on the array and we considered as significant, over-representation associated to a p-value lower than 0.05. Chromosome enrichment plots were produced with the UCSC genome browser (http://genome.ucsc.edu/cgi-bin/hgGateway).
